# Deregulation of exosomal miRNAs in rheumatoid arthritis patients

**DOI:** 10.1371/journal.pone.0289301

**Published:** 2023-07-27

**Authors:** Muhammad Zahid Hussain, Muhammad Shahbaz Haris, Muhammad Rizwan, Nida Sarosh Ashraf, Maryam Arshad, Ishrat Mahjabeen

**Affiliations:** 1 Department of Rheumatology, National University of Medical Sciences, Rawalpindi, Pakistan; 2 Department of Biosciences, Cancer Genetics and Epigenetics Lab, COMSATS University Islamabad, Islamabad, Pakistan; Stellenbosch University, SOUTH AFRICA

## Abstract

Exosomes are small-diameter endosomal vesicles secreted in all biological fluids and play biological/pathological roles in the cell. These pathological roles are played by exosome’s cargo molecules through inter-cellular communication. Exosomal cargo molecules contain proteins and miRNAs. miRNAs are small non-coding RNA fragments involved in the reduction of final protein output by destabilizing or suppressing the translation of target messenger RNA (mRNA). This deregulation of the protein due to miRNAs ultimately accelerates the process of disease pathogenesis. The role of exosomal miRNAs has been investigated in different diseases and the limited number of studies have been published concerning exosomal miRNAs and rheumatoid arthritis (RA). The current study is designed to investigate the role of exosomal miRNAs (miRNA-103a-3p, miRNA-10a-5p, miRNA-204-3p, miRNA-330-3p, and miRNA-19b) in the pathogenesis of RA. Furthermore, the role of selected exosomal miRNAs in RA pathogenesis was further explored by estimating oxidative stress and histone deacetylation in RA patients. In the current study, 306 RA patients and equal numbers of age/gender-matched controls were used. The level of expression of above-mentioned exosomal miRNAs was assessed by performing qRT PCR. Deacetylation and oxidative stress assays were performed to estimate the 8-hydroxydeoxyguanosine (8-OHdG level) and histone deacetylation levels using the Enzyme-linked immunosorbent assay (ELISA). Statistical analysis indicated a significantly downregulated expression of miRNA-103a-3p (p<0.0001), miR-10a-5p (p<0.0001), miR-204-3p (p<0.0001), miR-330-3p (p<0.0001) and miR-19b (p<0.0001) in RA patients compared to controls. Significantly increased levels of 8-OHd*G* (p<0.0001) and histone deacetylation (p<0.0001) were observed among RA patients compared to controls. Spearman correlation showed a negative correlation between the deregulated exosomal miRNAs and increased oxidative stress and histone deacetylation in RA patients. Receiver operating characteristics (ROC) curve analysis showed a good diagnostic specificity/sensitivity of the above-mentioned exosomal miRNAs among RA patients. These analyses indicated the potential role of deregulated exosomal miRNAs in the initiation of RA by targeting oxidative stress and histone deacetylation processes.

## Introduction

Rheumatoid arthritis (RA) is referred to as a common inflammatory joint disorder. The associated symptoms are inflammation and lifetime joint dysfunction and increased mortality rate [1]. The prevalence of RA in Western countries is approximately 1–2% while it appears to be 1% around the globe [2]. The estimated prevalence of RA is relatively higher in Northern Europe and the United States between 0.5 to 1% [3]. Among Asian countries, Pakistan faces a prevalence of about 0.5%. The evidence from the previous studies has demonstrated a prevalence of 0.142% in Karachi, predominantly among females [3].

Exosomes are nano-sized vesicles that may serve as a substantial biomarker in diagnosing auto-immune disorders. These vesicles possess natural characteristics such as biocompatible, less cytotoxic, non-immunogenic, long-lasting systemic circulation potential, and targeted delivery [4–7]. There is a high density of extracellular vesicles in synovial fluid of RA patients showing a substantial role in disease progression [8]. Thus, the diagnostic significance of exosomes in the blood is attributed to the onset and progression of joint deformities. In the current era, exosomes are of greater interest because of their therapeutic potential. Exosomes derived from stem cells can increase the repairing process of joints and delay the process of disease progression [9].

According to previous studies, metabolically engineered exosomes have altered surfaces for the direct reprogramming of macrophages [7–9]. Extracellular vesicles being released from the synovial fluid of RA patients’ joints control the propagation of joint disorder. Exosomes present in the synovial fluid are synthesized by infiltrated cells in the synovial joint. These include exosomes derived from platelets present in the synovial fluid of patients having moderate to severe inflammation of joints and rheumatoid arthritis [10,11]. However, there are evidence about the presence of exosome derivatives of granulocytes, neutrophils, and monocytes as well [1].

The cargo content present in the exosomes includes DNA, miRNA, and proteins. These exosomal cargo contents influence cell differentiation and viability, thus indicating a critical role in RA pathogenesis [11].

MiRNAs (miRs) are non-coding, conserved, single-stranded RNA of ~18–25 nucleotide long. These small entities are critical in regulating gene expression. miRNAs repress the gene expression by targeting and binding to the 3′-UTR sequence of their targeted mRNA. The silencing of gene expression involves either degradation of mRNA or destabilization of transcript resulting in suppression of target protein synthesis [12]. Exosomal miRNA-103a-3p, miRNA-10a-5p, miRNA-204-3p, miRNA-330-3p, and miRNA-19b have been observed deregulated in different diseases and found to be associated with an elevated risk of disease pathogenesis such as cancer [12–23]. Deregulation of these microRNAs has also been reported in joint inflammation by targeting different genes such as downregulation of miRNA-10a-5p increases the T-box transcription factor 5 activity and increase the demolition of cartilage [24]. Ding *et al*., (2022) has reported that miRNA-19b upregulation increases the occurrence of RA by downregulation the RAF1 expression and inhibiting the related pathways [25]. Zuo *et al*., (2015) have reported that miRNA-103a-3p controls bone development and inflammation by targeting the RUNX2 gene and acts as the first mechanosensitive microRNA to rescue osteoporosis [26]. Wang *et al*., (2022) has identified the structure-specific recognition protein 1 (Ssrp1) as the target site of the miRNA-204-3p, and deregulation of miRNA-204-3p results in excessive cellular proliferation and synovial inflammation and ultimately leads to RA [27]. Wu *et al*., (2021) have reported the change in the expression level of miRNA-330-3p in cartilage injury and bone deformities [28]. Previous studies have reported the involvement of miRNA-103a-3p, miRNA-10a-5p, miRNA-204-3p, miRNA-330-3p, and miRNA-19b in synovial inflammation, cartilage injury, and in inflammatory joint disorder [23–28]. No study has been published concerning involvement of exosomal miRNAs, miRNA-103a-3p, miRNA-10a-5p, miRNA-204-3p, miRNA-330-3p, and miRNA-19b in RA patients. So, the present study was designed for a more comprehensive understanding of the involvement of exosomal microRNAs in RA-related mechanisms such as development and the pathogenesis of RA. The expression levels of selected exosomal miRNAs were also correlated with oxidative stress and histone deacetylation of RA patients to figure out the mechanism behind the disease initiation and pathogenesis. Thus, the current research will pave the way in highlighting their potential role as a biomarker in joint abnormalities.

## Methodology

### Sample collection

The present study was conducted after prior approval from the COMSATS University Islamabad and military hospital Rawalpindi, Pakistan. 306 RA patients were recruited after confirmation by expert rheumatologists. Blood samples of equal number of age and gender-matched controls were also collected in the present study. Blood samples of RA patients and controls were stored at 4°C for further procedure. The demographic details of 306 RA patients and controls were given in Table 1. The inclusion criteria for the patients were confirmed RA diagnosis irrespective of their gender, age, or ethnic background. However, inclusion criteria for controls were age and gender-matched healthy individuals with no prior history of any type of RA and joint disorder.

**Table 1:** Demographic parameters of rheumatoid arthritis in study cohort.

Parameter	Patients	Controls
**Gender:** Male Female	179 127	165 141
**Age:** < 50 ≥50	141 165	149 157
**Anti-CCP:** Positive Negative	225 081	
**ESR:** < 31 ≥31	117 189	
**CRP:** <14 ≥14	90 216	
**Treatment:** Methotrexate Biologics	218 88	

ESR, erythrocyte sedimentation rate; CRP, C-reactive protein; Anti-CCP, Anti-cyclic citrullinated peptide.

The primary goals and objectives of the research were discussed with participating individuals and oral and written approval was attained from participants and their legal guardians, before collecting samples.

#### Ethical approval

The current research was performed after seeking prior approval by the Ethical Review Committee of the Military Hospital, Rawalpindi (RTMC # RHE-2019-124-87).

#### Exosome isolation and characterization

Exosomes were extracted from serum cells of RA patients and controls, using the ultracentrifugation method [29] and exosome extraction/purification kit (Thermo Fisher Scientific, CA, USA). The extracted exosomes were later characterized, using dynamic light scattering (DLS) and transmission electron microscopy (TEM) [29]. In TEM analysis, 5μl of exosomes sample was absorbed for 1 minute on carbon-coated grid. Exosomes were then stained with 0.75% uranyl formate for 20–30 seconds.

After removing the excess stain with filter paper, the grids were examined in a transmission electron microscope (JEOL 1200EX, USA) and images were recorded with an AMT 2k CCD camera (Thermo Fisher, USA).

Furthermore, exosomes extracted from serum samples of patients and controls were confirmed by *CD9*, *CD63*, and *CD81* antibodies. This confirmation was performed by commercially available *CD9*, *CD63*, and *CD81* ELISA kits (Abcam). Absorbance was estimated using a microplate reader (Platos R496, AMP Diagnostics), in which the calibration curves were obtained indicating the accurate sample calibrations.

#### RNA isolation and quantification

RNA was extracted from the exosomes using the Trizol extraction method as per the instruction of Mahjabeen *et al*., (2012) [30]. To avoid degradation, isolated RNA was stored at either -20°C for short-term storage or kept for long term usage. RNA integrity was later visualized on 1% TBE gel. The extracted RNA was quantified further using a Nanodrop spectrophotometer. RNA samples with an optical density (OD) ratio of 260/280 >1.65, were an appropriate quantity for synthesis of cDNA. The quality of extracted RNA was determined by 1% agarose electrophoresis. RNA bands were visualized on a UV trans-illuminator or Bio-Doc Analyzer.

#### cDNA synthesis and primer designing

The extracted RNA was converted to cDNA synthesis by a “High-capacity cDNA reverse transcription kit” (Invitrogen, USA). Synthesized cDNA was confirmed using β-actin. Firstly, *β-actin* primers were designed and optimized then PCR amplification was carried out on synthesized cDNA samples to confirm its presence. Then the amplified product was visualized on 2% agarose gel electrophoresis to confirm the specificity of the reaction.

The details of selected miRNAs, miRNA-103a-3p, miRNA-10a-5p, *miRNA*-204-3p, miRNA-330-3p, and miRNA-19b were retrieved from the miRNA database named miRBase. The Integrated DNA Technology primer quest tool was used for primer designing. Designed primer sequences were verified by NCBI Primer Blast and UCSC Insilco PCR. For designing β-actin and U6 primers sequences were retrieved from Ensemble Genome Browser while the rest of the procedure was same.

### Real-Time PCR

The Real-Time (RT) PCR also called qPCR was employed to evaluate the level of expression of selected miRNAs “miRNA-103a-3p, miRNA-10a-5p, miRNA-204-3p, miRNA-330-3p and miRNA-19b among samples of RA and controls. Furthermore, *U6* was used as the internal control. StepOne plus RT-PCR system (Applied Biosystems) was performed to carry out the qPCR reaction. The relative expression of miRNA-103a-3p, miRNA-10a-5p, miRNA-204-3p, miRNA-330-3p, and miRNA-19b was estimated using the 2^-ΔΔCT^ method at the mRNA level [30].

### Oxidative stress measurement and histone deacetylation in RA patients

Oxidative stress was measured by evaluating the 8-hydroxydeoxyguanosine (8-OHdG) levels in extracted exosomes from RA patients and controls by using a commercially available ELISA kit of 8–hydroxy 2 deoxyguanosine (Abcam, USA). A microplate reader was used to estimate the absorbance of *8-OHdG* (Platos R496, AMP Diagnostics, Austria). The accurate calibration of samples was illustrated through the calibrations of samples.

The histone deacetylation level from the extracted exosomes of the RA patients and controls was measured using the (HDAC) histone deacetylase activity assay kit (Abcam, USA). Extracted exosomes were treated with reagents of the assay kit. A microtiter plate Fluorescence Reader (Platos RII, AMP diagnostic, Austria) was used to measure the fluorescence intensity.

### Statistical analysis

The data were statistically analyzed using a statistical software tool i.e., Graph Pad Prism “Student t-test” and “one way ANOVA”. These statistical tests were used to evaluate the association between the expression level of selected exosomal microRNAs and clinicopathological parameters of RA patients. Spearman correlation analysis was performed to check the correlation between selected miRNAs and clinico-demographic parameters of RA patients. ROC curve analysis was performed to check the diagnostic value of miRNA-103a-3p, miRNA-10a-5p, miRNA-204-3p, miRNA-330-3p, and miRNA-19b in RA disease. The statistically significant value was adjusted at p ≤ 0.05. p < 0.05, p < 0.01, and p < 0.001 are indicated as (*), (**), (***) respectively.

## Results

### Characterization of exosomes

In the present study, exosomes were extracted from the serum of RA patients and controls. Extracted exosomes were characterized using modern characterization techniques including DLS, TEM, and ELISA.

**Fig 1:**
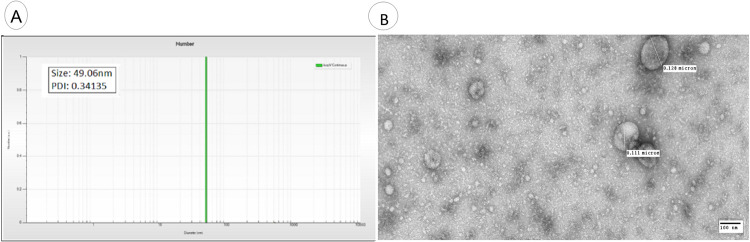
Characterization of exosomes extracted from RA patients and controls (A) Determination of exosomes size using DLS (B) Characterization of exosomes using TEM.

**Fig 2:**
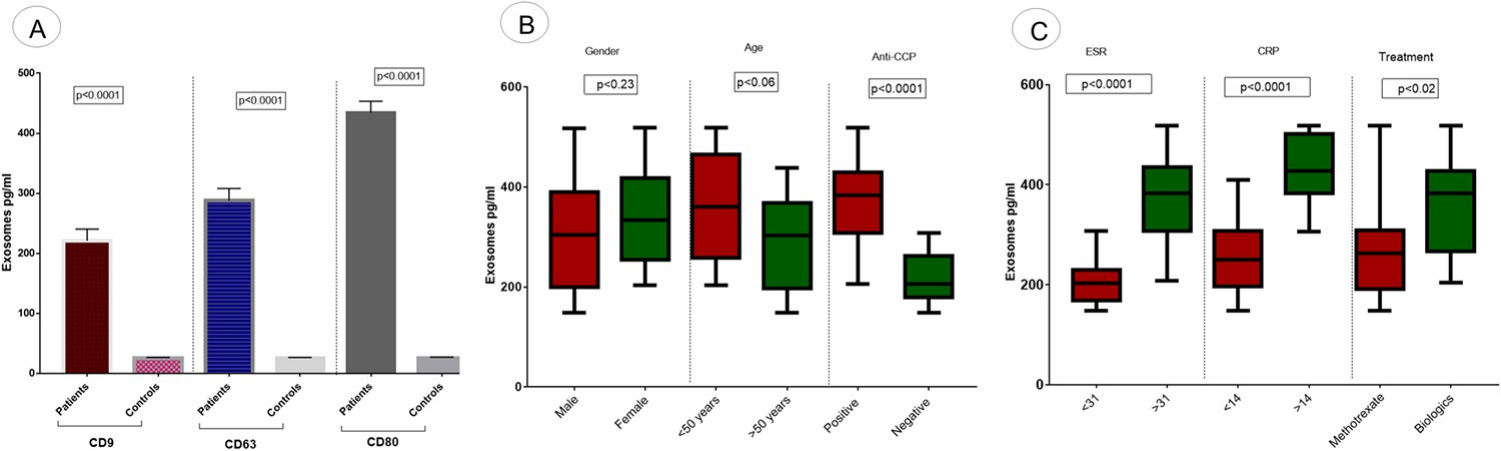
Expression of exosome surface markers. (A) Relative Expression of CD9, CD63, and CD81 in RA patients and controls. (B) Association of expression of exosome surface markers with demographic parameters of RA patients such as age, gender, and anti-CCP level. (C) Association of expression of exosome surface markers with pathological parameters of RA patients such as ESR level and CRP level and treatment. Pg/ml (picogram/mililitre).

DLS analyzed the exosomes by characterizing the average size distribution of the particles by particle size distribution. Extracellular vesicles having a range of 30-150nm were characterized as exosomes as illustrated in Fig 1A. The average size of extracted exosomes was observed as 49.06 nm whereas, the obtained polydispersity index (PDI) value was 0.34 as shown in Fig 1A. Exosomes were also characterized by TEM and shown in Fig 1B. In TEM, exosomes of different size were shown with size range of 30-150nm (Figs 1B and S1). ELISA was performed to investigate the expression of surface biomarkers of exosomes present in the serum of RA patients. Exosomes are rich in surface biomarkers CD8, CD63, and CD9. The optical density of surface markers was measured at 450nm as shown in Fig 2A. Exosome surface markers were observed significantly higher in RA patients compared to controls (p<0.0001; Fig 2A). The expression of exosome surface markers was also analyzed concerning different parameters of RA patients and shown in Fig 2. Exosomes surface markers were observed significantly higher in patients with positive anti-CCP (p<0.0001; Fig 2B) compared to negative anti-CCP, in patients with ESR >31 (p<0.0001; Fig 2C) compared to ESR <31, in patients with CRP >14 (p<0.0001; Fig 2C) compared to CRP<14, in patients receiving the biologics treatment (p<0.02; Fig 2C) compared to those receiving methotrexate treatment.

### Expression analysis of exosomal miRNAs

**Fig 3:**
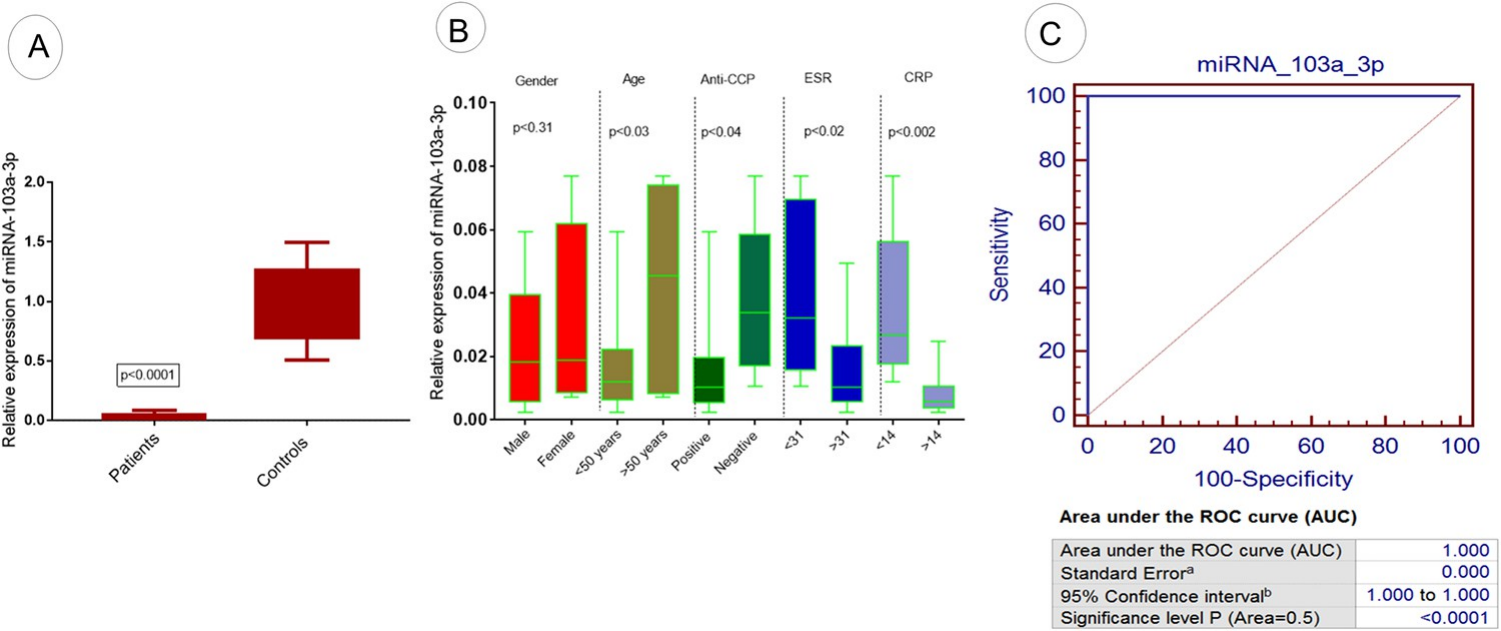
Expression analysis of exosomal miRNA-103a-3p in RA patients. (A) The relative expression level of miRNA-103a-3p in RA patients vs controls. (B) Association of relative expression of miRNA-103a-3p with demographic/pathological parameters of RA patients such as age, gender, anti-CCP level, ESR level, and CRP level. (C) ROC curve analysis of miRNA-103a-3p in RA disease. Level of significance p<0.05.

Expression analysis of miRNA-103a-3p, miRNA-10a-5p, miRNA-204-3p, miRNA-330-3p, and miRNA-19b was evaluated in RA patients and controls using qPCR. The results attained demonstrated a significant downregulation of miRNA-103a-3p in RA patients compared to controls (p <0.0001), as illustrated in the Fig 3A. Further analysis showed that significantly reduced expression of miRNA-103a-3p was observed in RA patients with age group <50 years (p<0.03) compared to patients with age group >50 years as shown in Fig 3B. Significant downregulation of said miRNA was also observed among the patients of RA with positive anti-CCP (p<0.04) compared to negative anti-CCP, with ESR >31(p<0.03) compared to ESR<31 and with CRP>14 (p<0.002) compared to CRP<14, as shown in Fig 3B. However, non significant downregulation of miRNA-103a-3p was observed in different gender groups of RA patients (Fig 3B)

**Fig 4:**
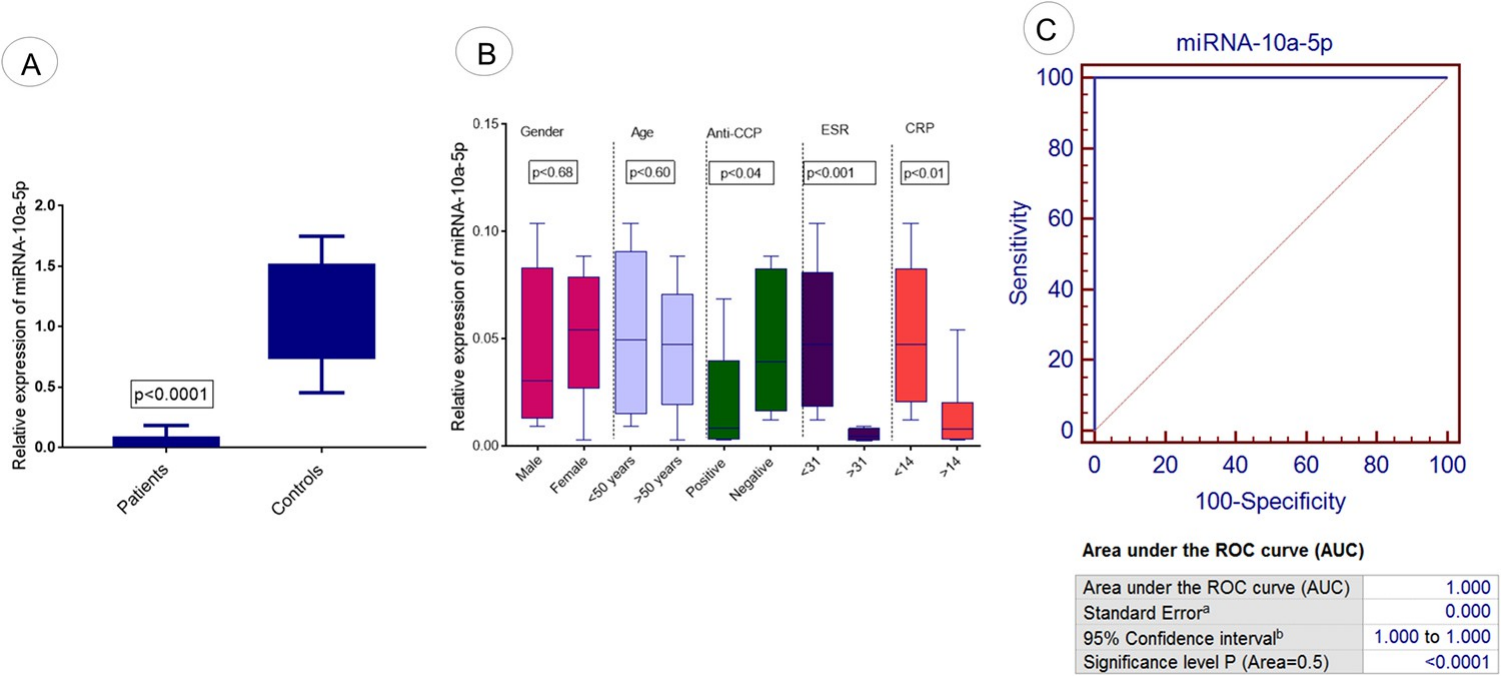
Expression analysis of exosomal miRNA-10a-5p in RA patients. (A) The relative expression level of miRNA-10a-5p in RA patients vs controls. (B) Association of relative expression of miRNA-10a-5p with demographic/pathological parameters of RA patients such as age, gender, anti-CCP level, ESR level, and CRP level. (C) ROC curve analysis of miRNA-10a-5p in RA disease. Level of significance p<0.05.

Expression analysis of miRNA-10a-5p was evaluated in RA patients and controls using qPCR. The results demonstrated a significant downregulation of miRNA-10a-5p in patients compared to controls (p <0.0001) as illustrated in Fig 4A. The expression level of said miRNA was correlated with different demographic parameters of RA patients. Significant downregulation of miRNA-10a-5p was also observed in RA patients with positive anti-CCP (p<0.04) compared to negative anti-CCP, with ESR >31(p<0.001) compared to ESR<31 and with CRP>14 (p<0.01) compared to CRP<14, as shown in Fig 4B. Furthermore, miRNA-10a-5p illustrated downregulation in different gender (p<0.68) and age groups (p<0.60) of RA patients.The results attained were statistically non-significant (Fig 4B).

**Fig 5:**
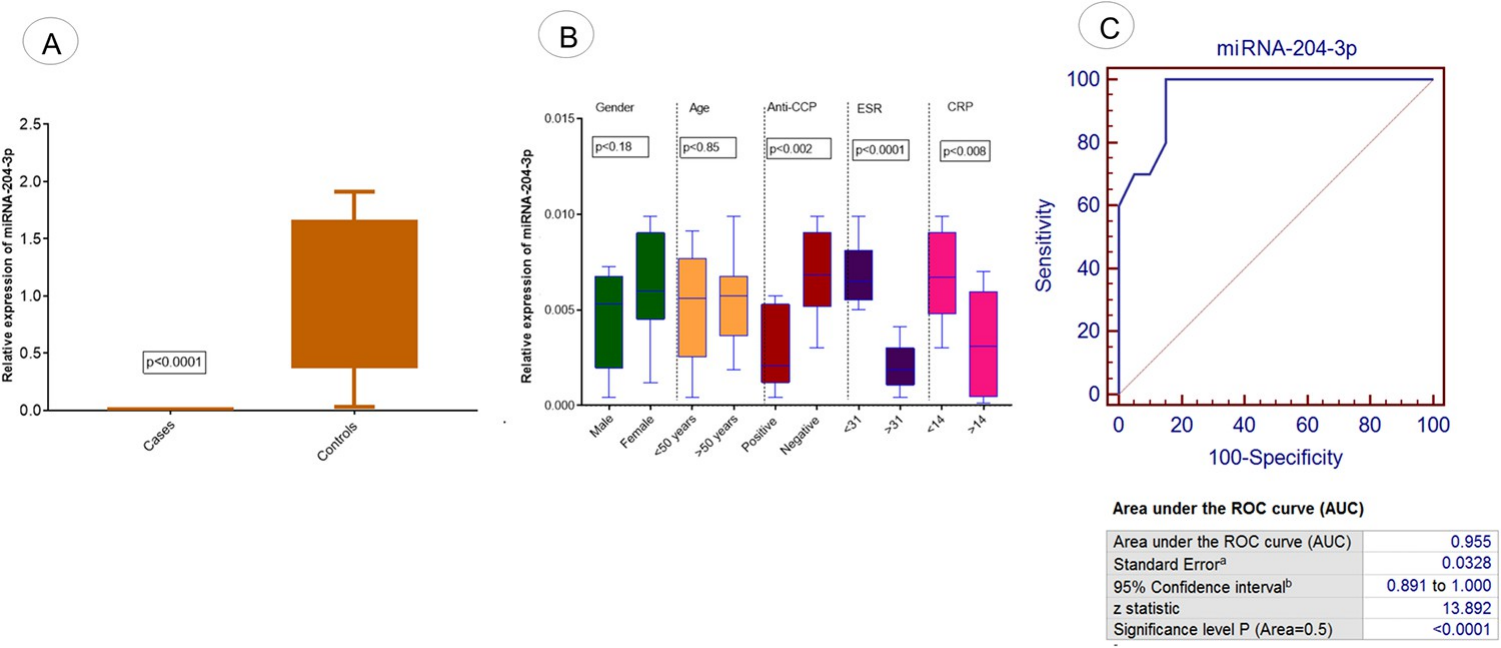
Expression analysis of exosomal miRNA-204-3p in RA patients. (A) The relative expression level of miRNA-204-3p in RA patients vs controls. (B) Association of relative expression of miRNA-204-3p with demographic/pathological parameters of RA patients such as age, gender, anti-CCP level, ESR level, and CRP level. (C) ROC curve analysis of miRNA-204-3p in RA disease. Level of significance p<0.05.

Expression analysis of third selected miRNA, miRNA-204-3p was evaluated in RA patients and controls, as shown in Fig 5. Significant downregulation of said miRNA was observed in patients (p<0.0001) compared to controls as shown in Fig 5A. The association of relative expression of miRNA-204-3p was assessed concerning different demographic parameters of RA patients. Significant downregulation of miRNA-204-3p was observed in R patients with positive Anti-CCP (p<0.002) and ESR >31 (p<0.0001) compared to patients with negative Anti-CCP and ESR <31, as shown in Fig 5B. Significantly downregulated expression of the miRNA-204-3p was observed in patients with CRP >14 (p<0.008) compared to patients with CRP <14, as shown in Fig 5B.

**Fig 6:**
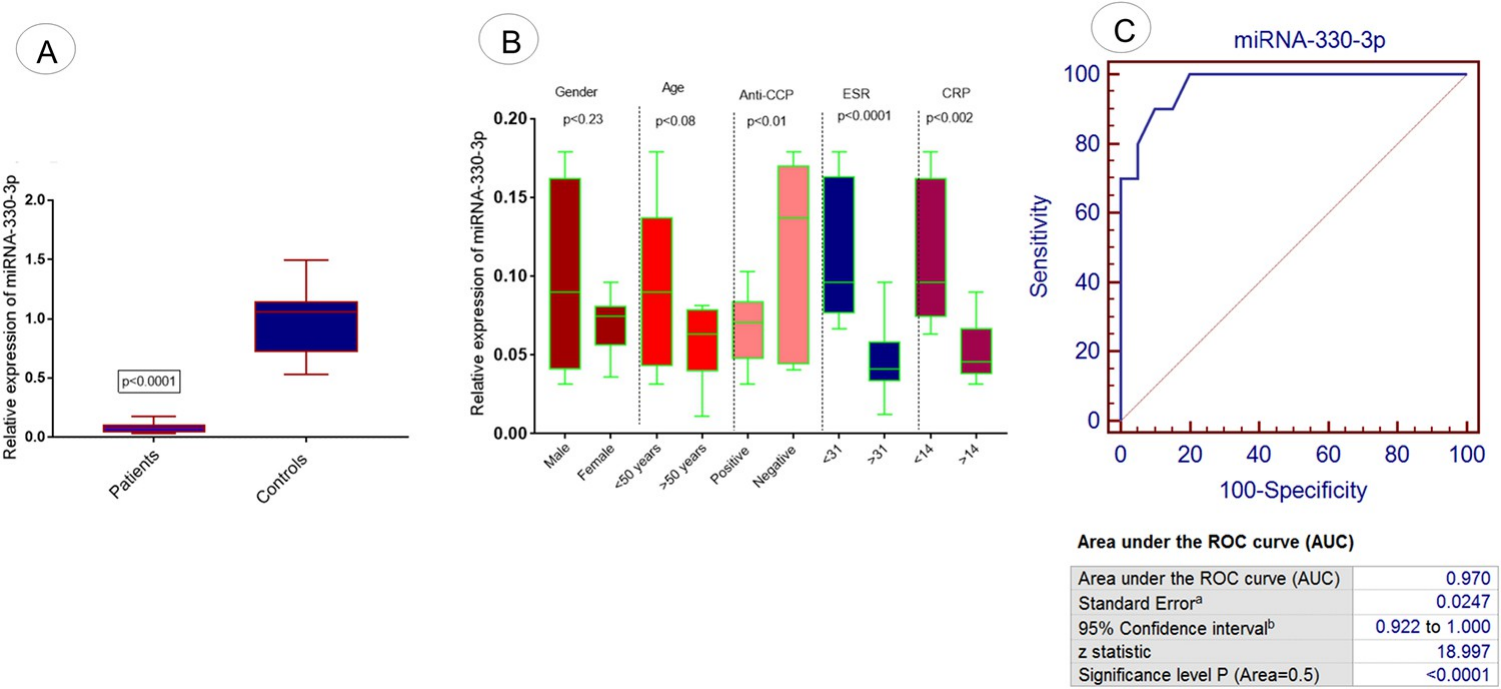
Expression analysis of exosomal miRNA-330-3p in RA patients. (A) Relative expression level miRNA-330-3p in RA patients vs controls. (B) Association of relative expression of miRNA-330-3p with demographic/pathological parameters of RA patients such as age, gender, anti-CCP level, ESR level, and CRP level. (C) ROC curve analysis of miRNA-330-3p in RA disease. Level of significance p<0.05.

Expression analysis of fourth selected miRNA, miRNA-330-3p was evaluated in RA patients and controls using qPCR. The results demonstrated a significant downregulation of miRNA-330-3p in RA patients compared to controls (p <0.0001) as illustrated in Fig 6A. The expression level of said miRNA was correlated with different demographic parameters of RA patients. Significant downregulation of miRNA-330-3p was observed in RA patients with positive anti-CCP (p<0.01) compared to negative anti-CCP, with ESR >31(p<0.0001) compared to ESR<31 and with CRP>14 (p<0.002) compared to CRP<14, as shown in Fig 6B. Howevernon significant downregulation of miRNA-330-3p was observed in different gender (p<0.23) and age groups (p<0.08) of RA patients (Fig 6B).

**Fig 7:**
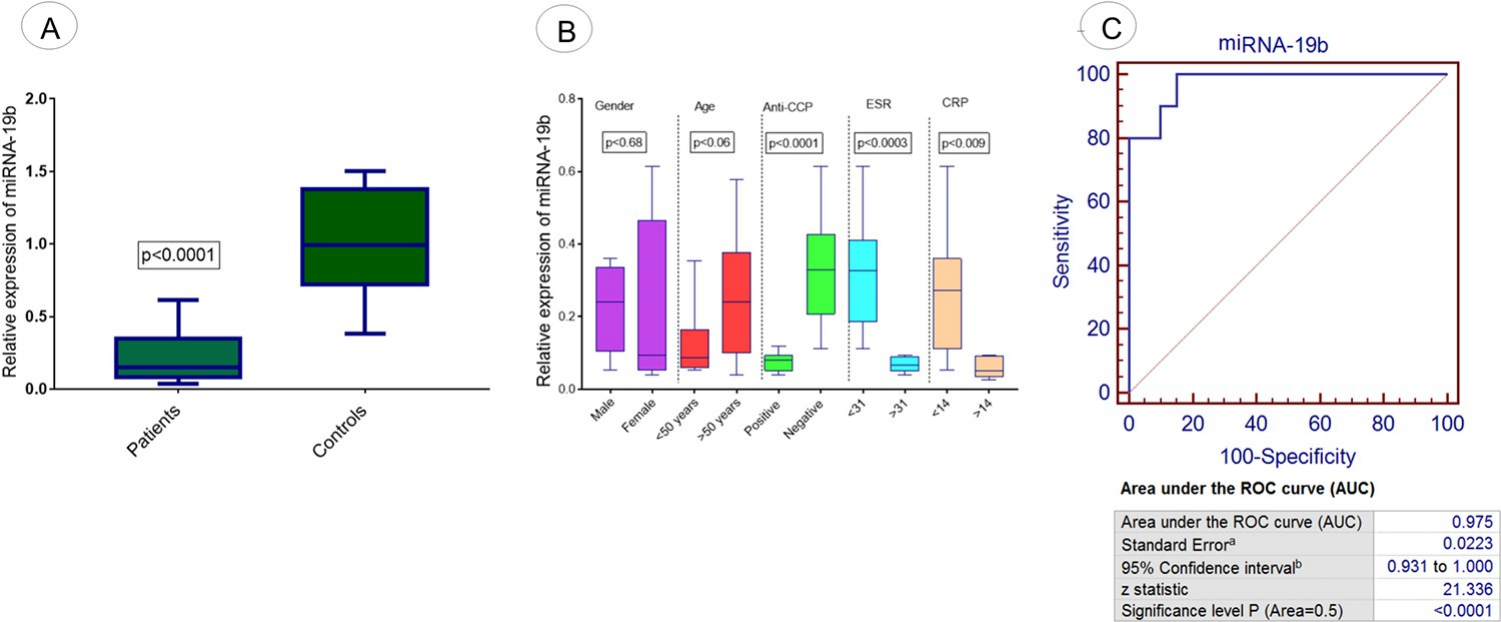
Expression analysis of exosomal miRNA-19b in RA patients. (A) Relative expression level miRNA-19b in RA patients vs controls. (B) Association of relative expression of miRNA-19b with demographic/pathological parameters of RA patients such as age, gender, anti-CCP level, ESR level, and CRP level. (C) ROC curve analysis of miRNA-19b in RA disease. Level of significance p<0.05.

Expression analysis of the fifth selected miRNA, miRNA-19b was evaluated in RA patients and controls using qPCR. The results attained demonstrated a significant downregulation of miRNA-19b in RA patients (p<0.0001) compared to controls as illustrated the Fig 7A. Expression analysis of miRNA-19b was evaluated in different demographic parameters of RA patients and controls. Significant downregulation of said miRNA was also observed in RA patients with positive anti-CCP (p<0.0001) to compared negative anti-CCP, with ESR >31(p<0.0003) compared to ESR<31 and with CRP>14 (p<0.009) compared to CRP<14, as shown in Fig 7 However, comparatively a non significant downregulated expression of miRNA-19b was observed in different age (p<0.06) and gender groups (p<0.68) of RA patients (Fig 7B).

**Fig 8:**
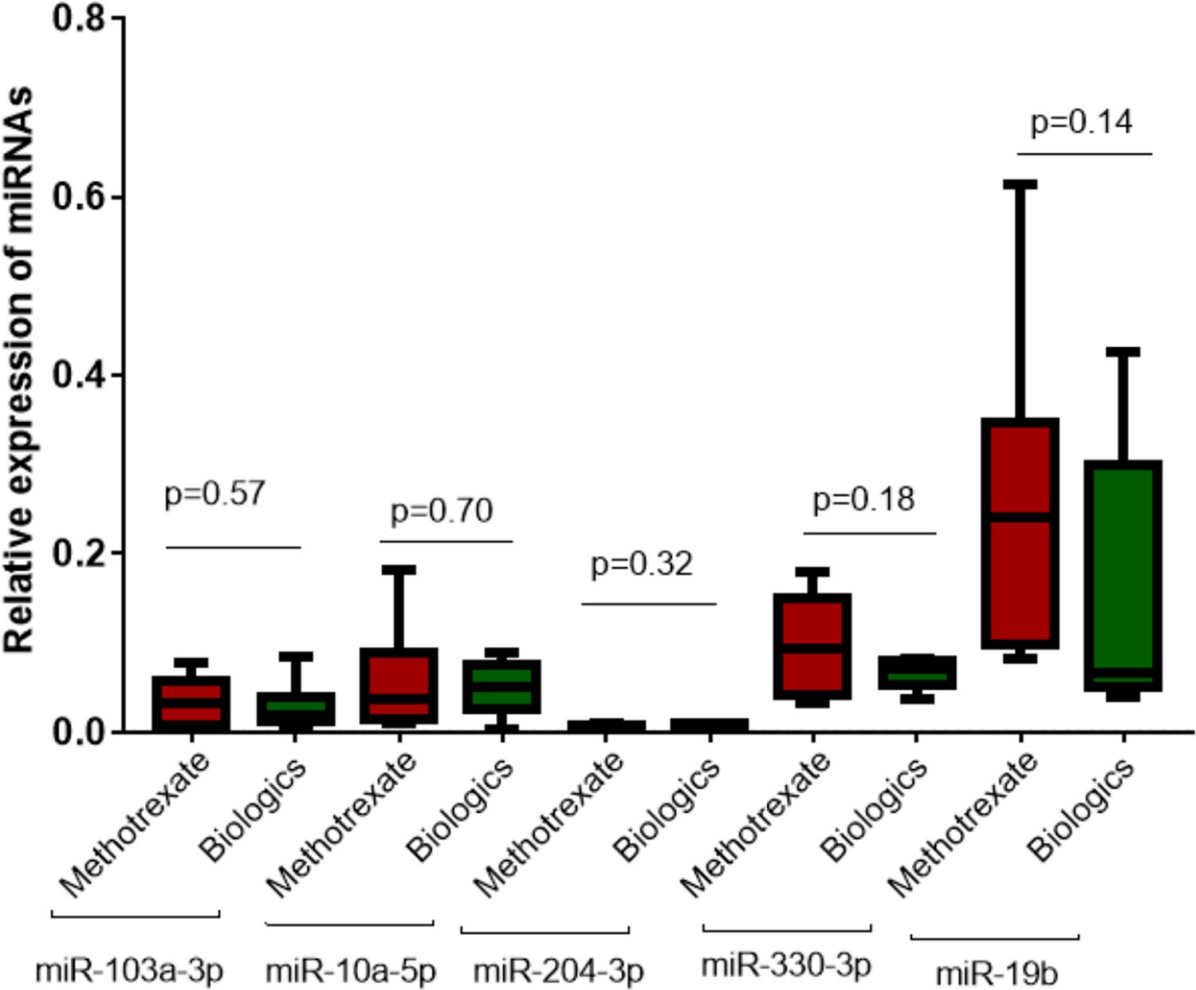
Association of treatment modalities such as methotrexate and biologics with relative expression of selected exosomal miRNA such as miRNA-103a-3p, miRNA-10a-5p, miRNA-204-3p, miRNA-330-3p, and microRNA-19b. Level of significance p<0.05.

Furthermore, RA patients were also divided into two categories based on treatments given to RA patients, as shown in Table 1. Expression levels of selected miRNAs were analyzed based on types of treatment such as methotrexate and biologics, as shown in Fig 8. No significant difference in the expression level of exosomal miRNAs was observed between both treatment modalities given to the RA patients.

### Diagnostic significance of selected miRNAs in RA patients

ROC Curve analysis was performed to detect and predict the diagnostic significance of miRNA-103a-3p, miRNA-10a-5p, miRNA-204-3p, miRNA-330-3p, and miRNA-19b. Analysis showed that 100% sensitivity and specificity were found in the case of miRNA-103a-3p (p<0.0001; Fig 3C) and miRNA-10a-5p (p<0.0001; Fig 4C). 97% sensitivity and specificity werefound in miRNA-330-3p (p<0.0001; Fig 6C) and miRNA-19b (p<0.0001; Fig 7C) and 95% sensitivity and specificity were observed in the case of miRNA-204-3p (p<0.0001; Fig 5C).

### Measurement of oxidative stress

**Fig 9:**
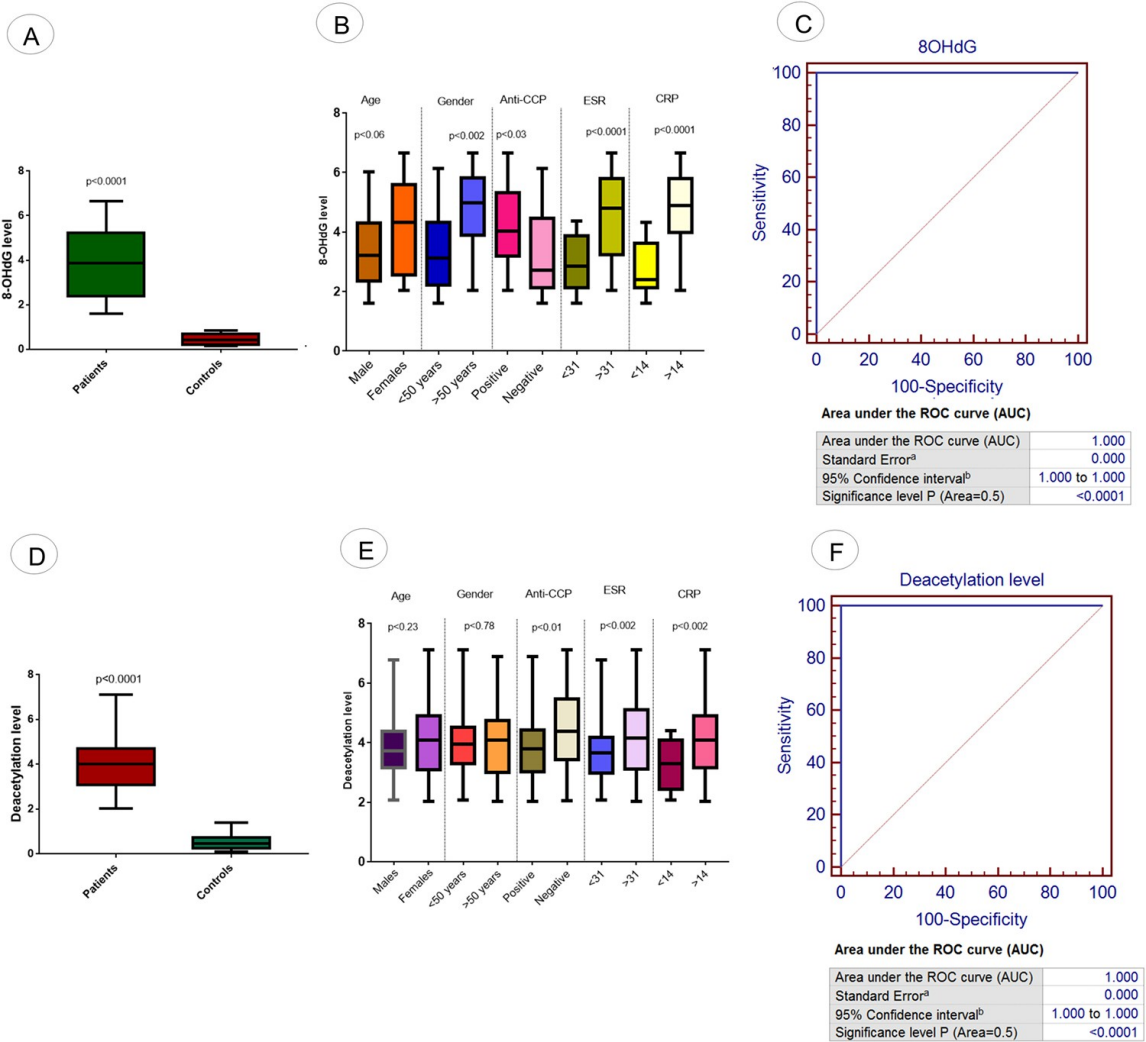
Measurement of oxidative stress and histone deacetylation in RA patients and controls. (A) 8-OHdG level in RA patients vs controls. (B) Association of 8-OHdG with demographic/pathological parameters of RA patients such as age, gender, anti-CCP level, ESR level, and CRP level. (C) ROC curve analysis of 8-OHdG in RA disease. (D) Histone deacetylation level in RA patients vs controls. (B) Association of histone deacetylation level with demographic/pathological parameters of RA patients such as age, gender, anti-CCP level, ESR level, and CRP level. (C) ROC curve analysis of histone deacetylation in RA disease. AUC = area under the curve; Level of significance p<0.05.

Oxidative stress was measured from the exosomes isolated from RA patients and controls as shown in Fig 9. Significantly increased level of 8-OHdG was observed in RA patients compared to controls, as shown in Fig 9A. This upregulation was obserpronounced in patients with positive anti-CCP (p<0.03) compared to negative anti-CCP, in ESR >31 (p<0.0001) compared to ESR<31 and in CRP >14 (p<0.0001) compared to CRP<14, as shown in Fig 9B. 8-OHdG level in RA patients was also calculatedconcerning the treatment modalities used for RA patients and shown in S2A Fig. A significant decrease level of 8-OHdG was observed in RA patients receiving biologics treatment (p<0.005) compared to patients receiving methotrexate treatment (SA Fig). ROC curve analysis was performed to assess the diagnostic significance of *8-OHdG* level in RA patients. AUC showed 100% specifcity and sensitivity of increased levels of *8-OHdG* in RA patients, as shown in Fig 9C.

### Epigenetic assessment of exosomes in RA patients

The epigenetic level of exosomes was measured among the RA patients and controls using histone deacetylation assay, as shown in Fig 9. Significant upregulation of histone deacetylation was found in RA patients (p<0.0001) compared to controls, as illustrated in Fig 9D. This upregulation was observed more pronounced in RA patients with positive anti-CCP (p<0.01) compared to negative anti-CCP, in ESR >31 (p<0.002) compared to ESR<31 and in CRP >14 (p<0.002) compared to CRP<14, as shown in Fig 9E. Histone deacetylation level in RA patients was also calculated concerning the treatment modalities used for RA patients and shown in S2B Fig. No Significant difference in histone deacetylation level was observed in RA patients receiving biologics treatment (p<0.15) compared to patients receiving methotrexate treatment (S2B Fig). ROC curve analysis was performed to assessed the diagnostic significant of histone deacetylation level in RA ptients. AUC showed the 100% specifcity and sensitivity of increased level of deacetylation in RA patients, as shown in Fig 9F.

### Spearman analysis of selected miRNAs

Correlation between selected miRNAs was observed using Spearman correlation as indicated in Table 2. A statistically significant positive correlation was found among miRNA-103a-3p vs miRNA-10a-5p (p<0.0001), miRNA-103a-3p vs miRNA-204-3p (p<0.0001), miRNA-103a-3p vs miRNA-330-3p (p<0.0001), miRNA-103a-3p vs miRNA-19b (p<0.0001), miRNA-10a-5p vs miRNA-204-3p (p<0.0001), miRNA-10a-5p vs miRNA-330-3p (p<0.0001), miRNA-10a-5p vs miRNA-19b (p<0.0001), miRNA-204-3p vs miRNA-330-3p (p<0.0001), miRNA-204-3p vs miRNA-19b (p<0.0001) and miRNA-330-3p vs miRNA-19b (p<0.0001) among RA patients, as listed in Table 2.

**Table 2:** Spearman correlation between exosomal miRNAs expression level and demographic parameters of rheumatoid arthritis patients.

Parameters	Gender	Anti-CCP	ESR	CRP	miRNA- 103a-3p	miRNA- 10a-5p	miRNA- 204-3p	miRNA-330-3p	miRNA-19b	8-OHdG	Deacetylation
Age	0.098	0.027	0.056	0.088	0.056	0.029	0.051	0.064	0.098	0.024	0.098
Gender		0.034	0.097	0.044	0.088	0.061	0.035	0.012	0.019	0.066	0.032
Anti-CCP			0.043	0.022	0.214*	0.239*	0.201*	0.340**	0.221*	0.16	0.17
ESR				0.219*	0.321**	0.309**	0.218*	0.616***	0.318**	0.236*	0.211*
CRP					0.440***	0.222*	0.234*	0.223*	0.290*	0.312**	0.412**
miRNA-103a-3p						0.617***	0.633***	0.688***	0.648***	-0.218*	-0.314**
miRNA-10a-5p							0.646***	0.692***	0.598***	-0.220*	-0.261*
miRNA-204-3p								0.655***	0.506***	-0.307**	-0.213*
miRNA-330-3p									0.599***	-0.614***	-0.204*
miRNA-19b										-0.222*	-0.264*
8-OHdG											0.355***

Spearman correlation coefficients. The p values were computed using the χ^2^-test.

* p< 0.05

** p< 0.01

*** p< 0.001.

A significant negative correlation was observed between miRNA-103a-3p vs 8-OHdG (p<0.03), miRNA-10a-5p vs 8-OHdG (p<0.03), miRNA-204-3p vs 8-OHdG (p<0.001), miRNA-330-3p vs 8-OHdG (p<0.0001), miRNA-19b vs 8-OHdG (p<0.03), miRNA-103a-3p vs histone deacetylation (p<0.001), miRNA-10a-5p vs histone deacetylation (p<0.02), miRNA-204-3p vs histone deacetylation (p<0.03), miRNA-330-3p vs histone deacetylation (p<0.03) and miRNA-19b vs histone deacetylation (p<0.02) in RA patients as shown in Table 2.

## Discussion

Rheumatoid arthritis(RA) is prevalent cause of disability, moratility, and morbidity around the globe. RA is referred as a common inflammatory joint disorder and symptomatically characterized by joint inflammation, tenderness and swelling with synovial joint destruction [2,3]. It is a multifactorial disease characterized by unknown etiologyy associated with complicated pathogenesis and severity [4,5]. The prevalence of RA in the United States and northern Europe is 0.5–1% [1]. Among Asian countries, Pakistan faces a prevalence of about 0.5%. The evidence from the previous studies have demonstrated a prevalence of 0.142% in Karachi predominantly among females [3].

Extracellular vesicles (EVs) displayed a high density in the synovial fluid of RA patients and were found associated with the progression of the disease [5]. EVs might serve as potential biomarkers for the treatment of rheumatic diseases [5]. Among EVs, small lipid bilayer extracellular vesicles known as exosomes, are secreted from maximum types of cells and can be identified in body fluids [31,32]. Exosomes play a vital role in communication between cells and act as vehicles for signal transmission and transfer of content, therefore regulating different pathological and physiological procedures of diseases [33,34]. RNAs (miRNA, mRNA, and long non-coding RNA), DNAs, proteins, and lipids are major constituents of exosome cargo. These constituents are useful only when they are transported to recipient cells [35–37]. Among the exosome cargo contents, miRNAs have been found involved in disease progression and development [38–40]. Most of the published studies on RA have focused on tissue/circulating miRNA resulting in a lack of knowledge regarding the involvement of exosomal miRNA as a biomarker for commonly occurring bone disease [41,42]. The scarcity of data concerning exosomal miRNAs has created a gap in the knowledge about the effective treatment and individual susceptibility to RA. The present study is designed to isolate and characterize the exosomes from RA patients and controls. Furthermore, five exosomal miRNAs were selected and the expression level of selected exosomal miRNAs was investigated among RA patients and controls. The level of expression of miRNAs was correlated with different pathological and demographic parameters of RA individuals.

In the present study, characterization of exosomes was performed using DLS and TEM. Sharp peaks were obtained having a diameter of approximately 100nm in diameter. Exosome extraction and purification are further confirmed by ELISA to confirm the presence of exosomal markers in RA patients and controls. The relative expression analysis of selected exosomal miRNAs (miRNA-103a-3p, miRNA-10a-5p, miRNA-204-3p, miRNA-330-3p, and miRNA-19b) showed significantly downregulated expression in RA patients compared to controls. Expression variations of selected exosomal miRNAs were correlated with demographic/pathological parameters of RA. Statistical analysis showed that this deregulated expression was found associated with aggressive behavior of RA such as positive anti-CCP, and increased ESR and CRP levels. These findings were with the previosly reported studies [43–45]. Several miRNAs are of great significance and serve as potent modulators of osteoarthritis(OA) and rheumatoid arthritis (RA). The potential role of miRNA-103a-3p in bone modulation has been studied extensively in many rheumatoid disorders[6]. Additionally, an upregulated expression of miRNA-103a-3p has been reported in RA patients and their asymptomatic first-degree relatives [7]. The expression level of miRNA-330-3p has been significantly upregulated among the intervention group compared to controls. The expression of miRNA-330-3p has been comparatively reduced in injured cartilaginous tissues [8]. The reason behind the deregulation of selected exosomal miRNAs and the increased risk of RA still needs to be explored. Few studies have reported the association between deregulation of exosomal miRNA and increased risk of disease. Chen et al., (2018) have reported that decreased expression of exosomal miRNA results in the induction of inflammatory reaction, increased RA aggressive properties, and angiogenesis [46]. Liu et al. (2021) have reported that exosomal miRNA depletion contributes towards a decrease in cell viability and increased in apoptosis of synovial fibroblast in RA patients [47]. Studies have reported that the deregulation of exosomal miRNAs results in an increased inflammatory response and an increased in oxidative stress which ultimately leads to increased bone disorder and arthritis [48,49].

In the second step of study, oxidative stress was measured in RA patients compared to controls. Analysis showed the significantly increased 8-OHdG level in exosome extracted from RA patients compared to controls. This upregulation was found linked with disease aggressiveness compared to early disease. The previously reported studies have shown link between the enhanced ROS level, decreased anti-oxidant activity, increased inflammation, and disease aggressiveness in RA patients [50,51]. Still, no study has been published regarding the level of 8-OHdG in exosomes extracted from the RA patients. Only surface thiol has been measured in exosomes extracted from patients having RA and a significantly reduced level of surface thiol was found in RA patients compared to controls [50,51]. Further analysis showed a significant neagtive correlation between the exosomal 8-OHdG level and exosomal miRNA. This showed that increased levels of exosomal 8-OHdG level result in deregulation of exosomal miRNA and increased disease severity/aggressiveness. Wang et al., (2018) have reported that an increased level of ROS in exosomes results in deregulated exosomal miRNAs and increased aggressiveness of disease. This increase promotes the activated neutrophils in synovial fluid of RA patient’s joint, which ultimately results in enhanced O_2_^_^ production and deregulation of the exosomal miRNAs [52].

To further elucidate the role of exosomal microRNA in RA pathogenesis, histone deacetylation levels were measured in extracted exosomes of RA patients and controls. Histone deacetylation level of exosomes was also correlated with the expression level of selected exosomal microRNAs. Previous studies have reported that miRNAs were found to directly link/target with histone deacetylation level and participate in chondrocyte proliferation/differentiation, osteoblast differentiation and pathogenesis of RA and OA [53,54]. Significantly increased level of histone deacetylation was observed in RA patients compared to controls and this deregulation was found associated with advanced stage of RA compared to the early stage. Furthermore, spearman correlation analysis indicated a significant negative correlation between the exosomal miRNA deregulation and deacetylation level. To date most studies regarding the correlation between the miRNAs and histone deacetylation have been focused on the miRNAs extracted from the blood samples of patients. Only a single study has reported the correlation between exosomal miRNA and histone deacetylation in chondrogenesis and cartilage degradation [55]. Mao *et al*., (2017) have reported the negative correlation between miRNAs expression and histone deacetylation level [55]. Saito *et al*., (2013) have reported that the use of histone deacetylation inhibitors results in decreased cartilage degradation and can act as a potential therapeutic agent for RA patient’s treatment [56]. Another study has reported that increased expression of HDACs leads to increased expression of miRNAs and activates interleukin-1 induced matrix metalloproteinase expression. These variations ultimately result in increased cartilage absorption and disease severity [57,58].

In conclusion, the current study showed potential deregulation of exosomal miRNAs (miRNA-103a-3p, miRNA-10a-5p, miRNA-204-3p, miRNA-330-3p, and *microRNA-19b*) in RA patients compared to controls. This deregulation indicated a strong correlation with more adverse stages of RA. Further analysis showed that deregulation of exosomal miRNA results in increased oxidative stress and increased histone deacetylation, which ultimately results in increased bone deregulation and disease severity in RA patients.

This study has the following potential limitations our study emphasize limited numbers of exosomal miRNAs with a smaller study cohort. Screening of different exosomal miRNAs and related pathway genes in larger study cohorts should be helpful to further illuminate the role of exosomal miRNAs in RA pathogenesis. Apart from expression analysis, *in-vitro* and *in-vivo* analysis of exosomal miRNAs should also be considered. The study may incorporate the use of other exosome characterization techniques such as nanoparticle tracking analysis (NTA) and western blot analysis.

## Supporting information

S1 Fig Transmission Electron microscopy of extracted exosomes from RA patients. Exosomes with different sizes are labelled with size range from 30-150nm.
(TIF)

S2 Fig Association of treatment modalities used in RA patients treatments such as methotrexate and biologics with (A) A) 8-OHdG level in RA patients, (B) Histone deacetylation level in RA patients. Level of significance p<0.05.
(TIF)
