# Supplementary figures and images for: Deregulation of exosomal miRNAs in rheumatoid arthritis patients
Source: PLoS One. 2023 Jul 27;18(7):e0289301. doi: 10.1371/journal.pone.0289301 (PMC10374114; doi:10.1371/journal.pone.0289301)

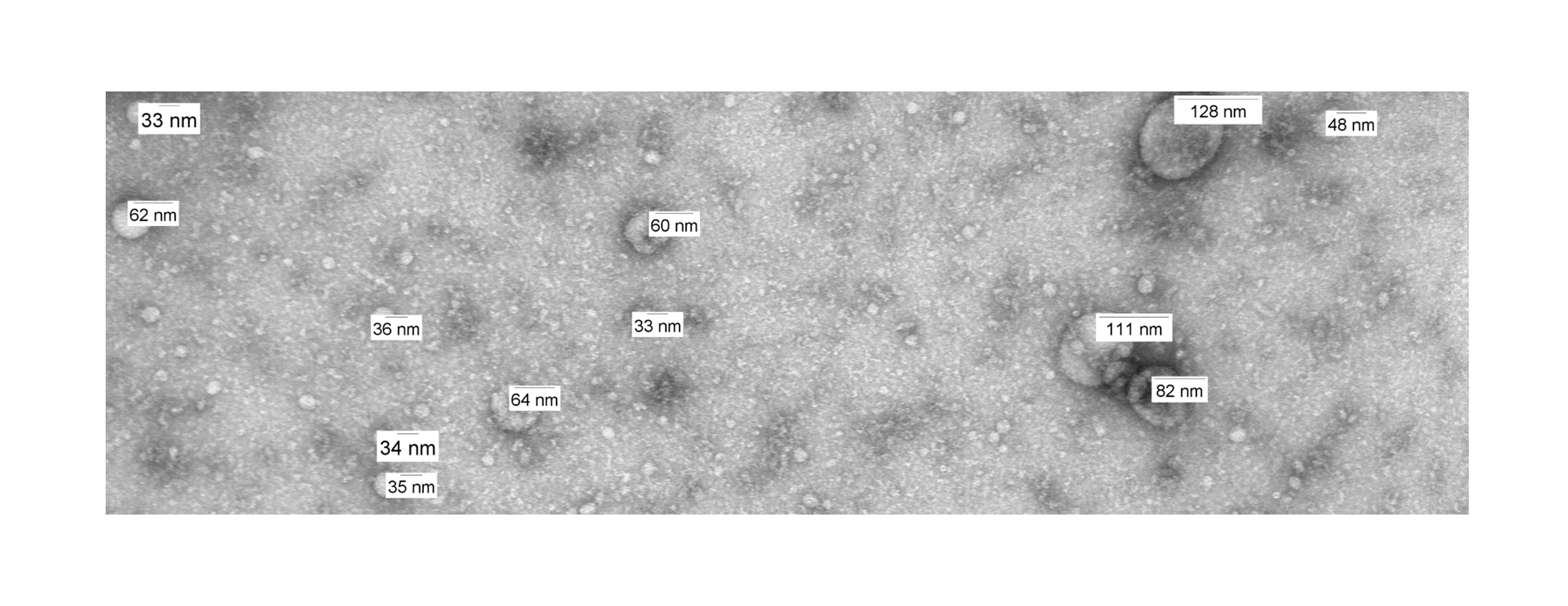

Supplement: S1 Fig — Exosomes with different sizes are labelled with size range from 30-150nm. (TIF) [file pone.0289301.s001.tif]

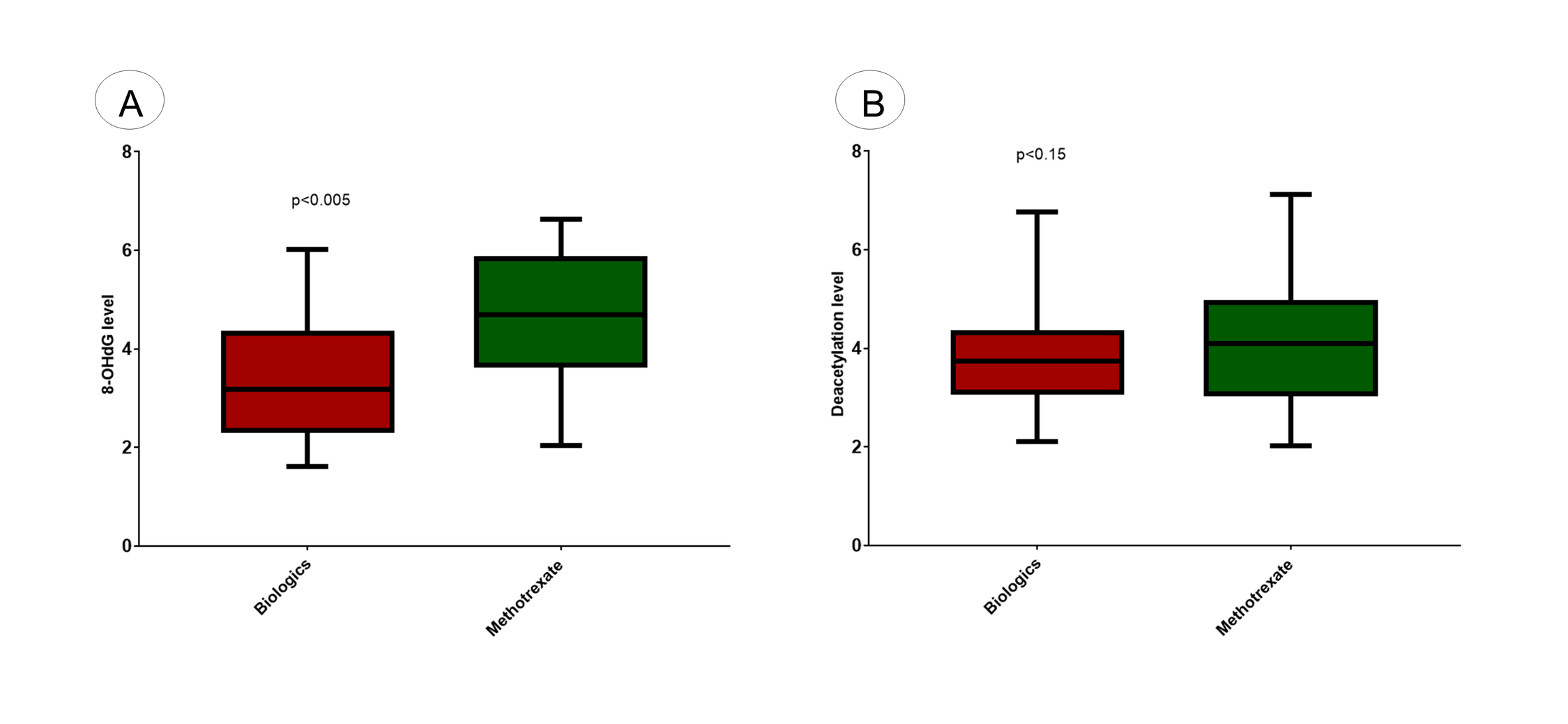

Supplement: S2 Fig — Association of treatment modalities used in RA patients treatments such as methotrexate and biologics with (A) A) 8-OHdG level in RA patients, (B) Histone deacetylation level in RA patients. Level of significance p<0.05. (TIF) [file pone.0289301.s002.tif]
